# Western gray whales on their summer feeding ground off Sakhalin Island in 2015: who is foraging where?

**DOI:** 10.1007/s10661-022-10022-x

**Published:** 2022-10-18

**Authors:** Lisa K. Schwarz, Glenn Gailey, Olga Tyurneva, Yuri Yakovlev, Olga Sychenko, Peter van der Wolf, Vladimir V. Vertyankin

**Affiliations:** 1 Ocean Sciences and Institute of Marine Sciences, University of California, Santa Cruz, CA 95060 USA; 2 Cetacean EcoSystem Research, Lacey, WA 98516 USA; 3 National Science Center of Marine Biology, Far East Branch, Russian Academy of Sciences, Vladivostok, 690041 Russia; 4 Sakhalin Energy Investment Company, Yuzhno-Sakhalinsk, Russia; 5 Kronotsky State Biosphere Reserve, Elizovo, Russia

**Keywords:** Habitat use, Prey energy, Sea of Okhotsk, *Eschrichtius robustus*, Photo-identification, Foraging

## Abstract

In the face of cumulative effects of oil and gas activities on the endangered western gray whale, informed management decisions rely on knowledge of gray whale spatial use patterns as a function of demographic group and prey energy. In particular, the gray whale foraging ground off Sakhalin Island consists of two distinct areas (nearshore and offshore) with the offshore feeding area exhibiting markedly high prey energy content. Based on photo-identification data collected from 2002 to 2015, we determined that gray whale use of the offshore feeding area increased with age. Pregnant females were more likely to be sighted only nearshore when nearshore prey energy and the proportion of nearshore energy from amphipods were higher. Likewise, females arriving with calves were less likely to be sighted offshore when the proportion of nearshore energy from amphipods was higher. Photo-identification effort in 2015 was increased substantially, with the intent of maximizing resighting data of individual whales to determine the relative proportion of different demographic groups utilizing the nearshore and offshore feeding areas. Comparing sighting data collected in 2015 with data from all previous years combined, mothers arriving with calves were sighted in the offshore feeding area earlier in 2015, with no evidence that they returned to forage nearshore later in the season. Other reproductive females constituted a higher proportion of the animals foraging nearshore prior to 2015, while juveniles were a higher proportion during 2015. Thus, the offshore feeding area is an important component of the gray whales’ annual life cycle, particularly if nearshore prey energy continues to decline, and offshore anthropogenic activities need to be monitored and addressed.

**Supplementary Information:**

The online version contains supplementary material available at 10.1007/s10661-022-10022-x.

## Introduction

The western or Korean–Okhotsk gray whale (*Eschrichtius robustus*) population forages off the northeastern coast of Sakhalin Island and has been of major conservation concern, particularly since the onset of oil and gas exploration and development activities close to their known foraging habitat. Gray whales observed off Sakhalin were thought to be a remnant population of whales previously considered to be extinct due to whaling (Bowen, 1974; Brownell & Chun, 1977). This population was thought to live solely in the western Pacific Ocean, foraging in the northern Okhotsk Sea in the summer and migrating to the southern China Sea to breed and reproduce in the winter (Reeves et al., 2008; Yablokov & Bogoslovskaya, 1984). Following their rediscovery in the Sakhalin area in the mid-1970s, the western gray whale population was listed under Category 1 as “on the verge of extinction” in the Russian Federation Red Book and Critically Endangered by the International Union for the Conservation of Nature (IUCN) (Brownell & Chun, 1977; Hilton-Taylor, 2000; Reilly et al., 2008; Weller et al., 2002a). While little was known about this population in the mid-1990s, extensive research over the past two decades has increased our knowledge about their site fidelity, genetic relatedness to eastern gray whales, migration patterns, and population size, dynamics, and structure (Brüniche-Olsen et al., 2018; Cooke, 2010; LeDuc et al., 2002; Mate et al., 2015; Tyurneva et al., 2018; Yakovlev et al., 2011). Based on an assessment of this body of available information, as well as a decision to include additional gray whales observed off Kamchatka, the IUCN re-classified the western gray whale as Endangered (Cooke et al., 2018). Nevertheless, the number of western gray whales foraging off Sakhalin Island remains very small, with approximately 175 individuals over the age of one year, of which 33 are known reproductive females (Cooke et al., 2017).

**Fig. 1:**
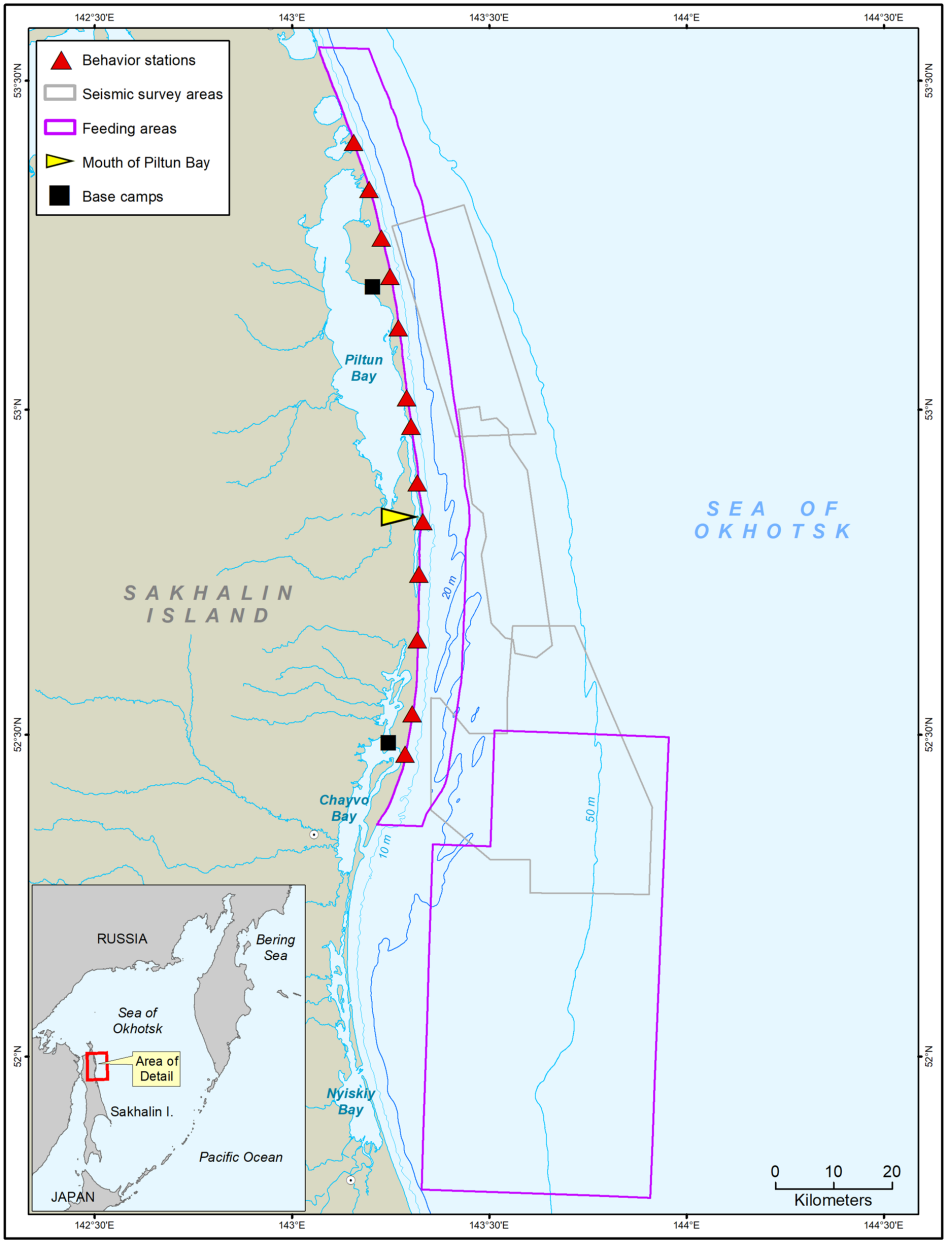
Study area with nearshore and offshore feeding areas (pink outline), behavior stations (red triangles, base camps (black squares), seismic survey areas (gray outline), and mouth of Piltun Bay (yellow arrow)

The gray whale foraging ground off Sakhalin Island consists of two distinct areas (nearshore and offshore) that are spatially small compared to the eastern gray whale foraging grounds in the northern Bering and Chukchi Seas (Mate et al., 2011; Moore et al., 2003) (Fig. 1). The two Sakhalin feeding areas are rich in benthic prey resources, particularly amphipods (Blanchard et al., 2019; Fadeev, 2011), which contain high energy density and are considered to be the whales’ preferred prey (Darling et al., 1998; Kim & Oliver, 1989; Maresh et al., 2022; Nerini, 1984; Pike, 1962). Photo-identification studies, conducted since 2002, and genetic sampling have led to new findings about site fidelity, general movement patterns, and age, sex, and reproductive status of some animals (Bröker et al., 2020; Tyurneva et al., 2018). For example, individuals have been observed moving between the nearshore and offshore feeding areas within a foraging season. However, mother–calf pairs and recently weaned calves appear to be limited to the shallow waters of the nearshore feeding area (Bröker et al., 2020; Tyurneva et al., 2010, 2018).

Despite the considerable amount of research, many questions remain relative to individual intra-seasonal movement patterns and habitat use for the different demographic groups and by age, particularly with respect to prey energy. Such information is valuable for management decisions regarding oil and gas activities that have the potential to disturb gray whales during their foraging season. Although there is no evidence of direct mortality due to oil and gas activities off Sakhalin Island, such activities could reduce a whale’s ability to optimally forage, which could ultimately affect life functions, such as reproduction and survival that could lead to population-level effects (Villegas-Amtmann et al., 2017). Sound exposure from seismic surveys has elicited behavioral and distributional responses in gray whales (Gailey et al., 2007; Weller et al., 2002b; Yazvenko et al., 2007). Mitigation measures were aimed at reducing temporal and spatial overlap between oil and gas exploration activities and gray whales, especially for animals foraging nearshore, with the goal of minimizing potential disruption in gray whale foraging as well as preventing auditory injury from airguns (Bröker et al., 2015; Johnson et al., 2007; Aerts et al., 2022). An enhanced understanding of intra-seasonal habitat use patterns for different demographic groups could further reduce exposure, particularly for individuals or groups of individuals that may be more restricted in their foraging opportunities or particularly sensitive to exposure, such as younger individuals (calves and juveniles) and reproductive females.

The objective of this paper was to understand how gray whales of different age and demographic groups used the nearshore and offshore feeding areas both within and between foraging seasons. Specifically, we examined the annual probability of sighting location (nearshore only, offshore only, or both) as a function of age and prey metrics. Given the high energetic costs of reproduction, we specifically analyzed the annual probability of sighting location as a function of prey for successfully pregnant females, i.e., those that successfully returned to the feeding area with a calf the following year, and for females with calves. Photo-identification surveys have been conducted since 2002, and the data collection effort in 2015 was increased substantially with the intent of understanding intra-seasonal changes in gray whale habitat use (Aerts et al., 2022). Therefore, we estimated the demographic makeup of animals identified nearshore as a function of day of year, comparing results from 2015 with the combined 2002–2014 dataset.

## Materials and methods

### Foraging habitat and study area

Photo-identification (photo-ID) studies were conducted along the northeastern coast of Sakhalin Island, Russia, within the two known gray whale foraging areas (Fig. 1). The nearshore feeding area generally extends out to the 20 m contour (~ 5 km from shore) and approximately 120 km along the shore of Piltun and Chayvo Bays (Aerts et al., 2022). Gray whales were first discovered feeding offshore of Chayvo Bay in waters of 35 to 60 m (the offshore feeding area) in 2001 and have been observed there during line-transect surveys each subsequent year (Meier et al., 2007; Yakovlev et al., 2009).

For the purposes of this study, we categorized photo-ID gray whale sighting and benthic data as nearshore or offshore based on study area boundaries from other studies (Maresh et al., 2022; McHuron et al., 2021). The boundaries of the two study areas were determined using a combination of the long-term benthic sampling grid and whale sightings in the area over time. Data within 8 km of the shoreline were considered nearshore. Sightings during photo-ID surveys and benthic data between 51.78˚–-52.50˚ N and 143.33˚–143.94˚ E, with the exception of the northwest corner, were categorized as offshore (Fig. 1).

### Data collection

Gray whale photo-ID data were collected annually from 2002 to 2015 utilizing various combinations of five different types of platforms: a large vessel and a small boat launched from the large vessel (2002–2015), a small inflatable boat launched from shore (2013–2015), one to two mobile shore-based teams driving along the coastline (2013–2015), and one to five stationary shore-based teams that collected photo-ID data in conjunction with gray whale behavior data (2004–2010 and 2015). The large vessel and small boat covered both the nearshore and offshore areas. Shore-based photo-ID efforts covered only the nearshore area, with the zodiac limited to the region around the Piltun Bay mouth. In general, while effort was high, environmental conditions and logistical constraints prevented consistency in photo-ID data collection from day to day both temporally and spatially.

Photographs in the nearshore and offshore areas were taken from the large vessel when the vessel was involved in data collection for other studies (benthic, acoustic, distribution) or when whales opportunistically approached within a reasonable distance from the vessel. When larger numbers of whales were seen and environmental conditions allowed safe operations, the small boat was launched from the vessel. For efficiency, boat launches from the large vessel were focused in areas of high whale density. Photographs of whales in the offshore area have generally been more difficult to collect due to larger swell and less predictable gray whale movements after deep dives.

Photo-ID data collection by shore-based, mobile teams took place during good weather conditions with visibility of at least 500 m and maximum Beaufort sea state of 5 (Yakovlev et al., 2015). Onshore, vehicle-based photo-ID teams sought to combine an opportunistic strategy with a consistent path while covering the whole shoreline during a workday. The teams were stationed both north and south of the mouth of Piltun Bay (Fig. 1). Teams started their surveys near their camp location but would choose to execute their survey either in a north–south or south-north pattern depending on tide, time of day, and weather. During the survey, teams tried to locate gray whales from the vehicle moving at a constant speed of 25–30 km/h. Prior to 2013, frequent stops were made at specific locations to scan for whales.

Photo-ID data collection by shore-based behavior teams was either opportunistic during behavior data collection, when a whale was close enough to photograph, or when behavior data collection was not possible (Beaufort sea state 4 +). In the latter case, rather than remaining stationary, observers would sometimes walk or drive along the coastline to collect photo-ID data.

Photographers attempted to capture five aspects of the whales’ bodies: left and right sides, head, peduncle region, and flukes. However, some body parts are more difficult to capture, so priority was given to photographing the right and left sides of each whale. In some cases, video imagery was used as a backup to photography collected from vessels. To identify individual whales, standard photo-recognition methods were used (Hammond et al., 1990), with modifications developed specifically for gray whales (Calambokidis et al., 2002). Individual gray whales were identified using unique patterns of permanent body pigmentation, with scars and barnacle patches to augment the matching process. Photo-ID data were processed with the Discovery Photo-ID data management system (Gailey & Karczmarski, 2012).

Blanchard et al. (2019) describe benthic prey sampling surveys and biomass estimates in more detail. In brief, benthic biomass data were collected using a van Veen grab deployed in a maximum of 72 nearshore and 48 offshore cells annually between 2002 and 2014. Some design variations occurred between years (Fadeev, 2002, 2005, 2007a, b). In particular, more spatially and temporally detailed sampling took place in 2015 within the nearshore area (Blanchard et al., 2022). In 2015, additional samples were collected to determine energy content of common gray whale benthic prey (Maresh et al., 2022).

### Analyses

Photo-ID data used for the analysis included 7881 sightings with geographic positions located within the defined nearshore and offshore study areas (Fig. 1). An additional 853 sightings with no positional information were included in the analysis because the general feeding area (nearshore or offshore) was known. Since calves arrive at the foraging grounds roughly 6 months after birth, ages were defined as 1 year increments starting at 0.5 years old and ending at the maximum known age of 12.5 years old. When known, animals were placed in demographic categories: calves, mothers with calves, other known reproductive females (previously seen with calves), males more than 4.5 years old, and juveniles 1.5 to 4.5 years old. Sighting days were defined as days when whales were sighted.

To quantify the relationship between foraging area and benthic prey, three different annual prey metrics were calculated, using location data to categorize each biomass grab sample as nearshore or offshore, focusing on six primary prey species (Amphipoda, Bivalvia, Cumacea, Isopoda, Polychaeta, and Actinopterygii (primarily sand lance)). For comparable inter-annual values, benthic samples systematically collected in predefined grids in years 2002–2010 and 2013–2015 were used (Blanchard et al., 2019). Summing biomass values for the six prey species groups for each grab, the annual mean biomass per grab both nearshore and offshore was calculated. Extending the methods described in Maresh et al., (2022), the mean annual benthic prey energy per square meter was determined by multiplying biomass values by the distribution of energy content for each species group. The total energy distribution was calculated by summing the energy content for all six species groups for each sample grab. Since gray whales may prefer amphipod prey, the proportion of energy from amphipods was also calculated. Distributions of energy and proportion energy from amphipods for each grab were combined by year to determine nearshore and offshore mean amphipod proportions and prey energy.

Multinomial logit regression was used to determine how annual sighting location (*L*_*i*_, where *i* = nearshore only (*N*), both nearshore and offshore (*B*), or offshore only (*O*)) changed as a function of eight different variables: age (*a*), relative effort (number of nearshore days/number of offshore days, *D*), nearshore and offshore prey biomass (*B*_*n*_ and *B*_*o*_), nearshore and offshore prey energy (*E*_*n*_ and *E*_*o*_), and nearshore and offshore proportion of total energy from amphipod energy (*A*_*n*_ and *A*_*o*_):

1 $$logit\left({L}_{i\ne N}\right)={\beta }_{0i}+{\beta }_{1i}x$$

where $${L}_{N}=1.0-{L}_{B}-{L}_{O}$$.

Predictor variables were tested in separate models, using Bayesian model averaging to determine the best model fit (discrete model expansion). We also used discrete model expansion to determine the annual proportion of successfully pregnant females in the three sighting categories as a function of annual relative effort and the six prey variables. The analysis was done for the season of pregnancy and the following year when the female arrives with a calf and subsequently weans it.

To understand how demographic groups use the two feeding areas during a season, we likewise used multinomial logit multivariate regression to quantify the proportion of nearshore observed animals by demographic category over the foraging season (*D*_*j*_ where *j* = calves, mothers with calves, other reproductive females (*OMF*), juveniles, or males > 4.5 years old (*M*)):

2 $$logit\left({D}_{j}\right)={\beta }_{0j}+{\beta }_{1j}t+{\beta }_{2j}{t}^{2}+{\beta }_{3j}{t}^{3}$$

The continuous model expansion was performed for 2015 and 2002–2014 for the nearshore area. Proportions for the demographic group with the least data were defined by proportions of the other groups. For 2015, ${D}_{M}=1.0-{\sum }_{j\ne M}{D}_{j}$, and ${D}_{OMF}=1.0-{\sum }_{j\ne OMF}{D}_{j}$ for earlier years. Offshore sample size was too small for statistical analysis, so general results along with proportion data across the seasons are provided.

The resulting proportions from the above analyses do not represent the absolute proportions of different demographic groups in the population. For example, males were almost exclusively identified genetically and therefore most likely underrepresented in the data since not all animals have been genetically identified. In addition, mother–calf pairs generally occur closer to shore and, thus, have a higher probability of identification by shore-based teams (and better quality photographs for identification). Vessel-based teams were more likely than shore-based teams to identify animals in the nearshore feeding area that occur farther from shore (> 1 km).

To compare reproductive rates of nearshore-only females to females that use both nearshore and offshore areas, the mean inter-calf intervals for each individual were calculated. A two-sided *t* test on the log of the means was performed, assuming unequal variances between the groups, using the t.text function in program R (R_Core_Team, 2020). The inter-calf interval is most likely an underestimate of reproductive rate because the mothers’ identities have not been determined for all calves. We assume the bias is equivalent for the two groups of reproductive females.

Bayesian R packages “brms” and “rjags” were used to estimate posterior distributions of regression parameters and to determine the best-fitting models (Bürkner, 2017, 2018; Plummer, 2019) (Online Resource 1). Priors on parameters were uniform with boundaries wide enough that posterior distributions of the parameters were not truncated at any prior boundaries. Standard practices (multiple independent chains with low lag-1 autocorrelation) ensured mixing, convergence, and stationarity in posterior samples using R packages “MCMCvis,” “coda,” and “bayesplot” (Gabry & Mahr, 2021; Gabry et al., 2019; Plummer et al., 2006; Youngflesh, 2018). To analyze sighting location as a function of age, effort, and the six prey metrics, Bayesian model stacking was used to average over multiple models when no one individual model was weighted > 0.9 (Yao et al., 2017).

To quantify the proportion of different demographic groups in the nearshore area over time, each demographic group was analyzed separately using logistic regression. The method allows for different demographic group proportions to change in different ways as a function of time. Using the R package “loo,” leave-one-out analyses determined the best-fitting model for each demographic group (Vehtari et al., 2020). When differences between two models with the highest expected log pointwise predictive density were < 1.0, we selected the model with fewer parameters.

## Results

### Annual sighting summary

**Fig. 2:**
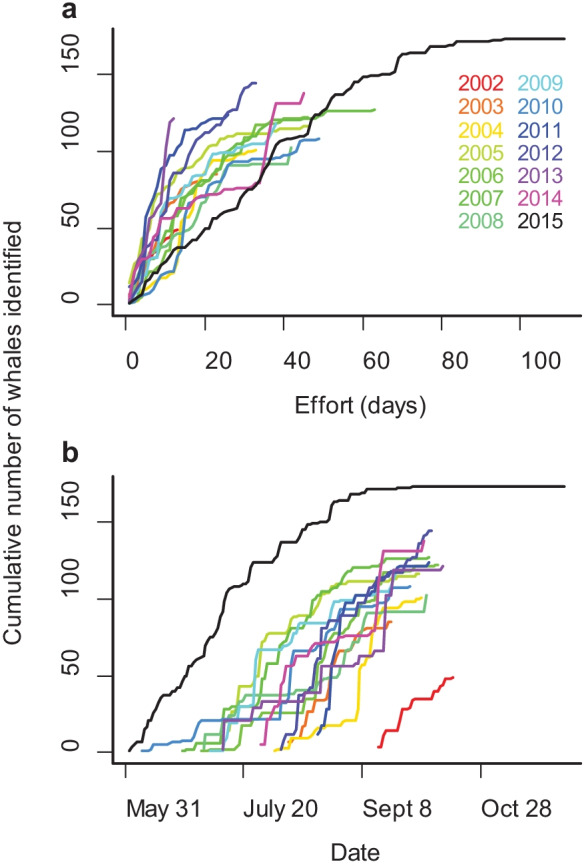
Cumulative number of individuals identified each year by **a** number of days with whale sightings and **b** day of year. Photo-identification surveys depend on weather conditions and are therefore not conducted on every day of a field season. Survey start dates also vary by year, so graphs are not identical

The number of sighting days and length of data collection period in 2015 were higher than other years, most notably for the nearshore feeding area (Table 1, Fig. 2a). Due to the increased number of teams operating from shore, the number of nearshore sighting days in 2015 was almost twice the maximum number of sighting days recorded for the previous 13 years (110 vs. 58 days). In 2015, a total of 173 whales were identified within the study area, the most whales identified in a single season over the 14-year period (Table 1). While within-day resightings were similar across years, 2015 represented a year with the highest resighting rates across the season. Ninety-four percent of individuals were seen on more than one day in 2015, with one whale seen on 42 days.

**Table 1:** Annual photo-identification sighting statistics for gray whales observed within the defined nearshore and offshore feeding areas off Sakhalin Island

	Year													
	2002	2003	2004	2005	2006	2007	2008	2009	2010	2011	2012	2013	2014	2015
Number of days with sightings nearshore	5	14	31	41	43	58	38	37	48	21	19	10	44	110
Number of days with sightings offshore	8	11	4	5	6	14	6	3	5	6	15	2	5	22
Proportion of animals sighted more than once on the same day	0.22	0.25	0.27	0.22	0.21	0.22	0.15	0.30	0.20	0.29	0.32	0.35	0.31	0.29
Average number of times an animal was sighted in 1 day	1.26	1.34	1.34	1.34	1.23	1.29	1.16	1.40	1.28	1.36	1.51	1.51	1.40	1.40
Maximum number of times an animal was sighted in 1 day	3	5	5	9	3	5	3	5	8	4	7	7	5	7
Total number of animals ID’ed in a season	49	85	101	116	122	127	102	118	108	124	144	121	138	173
Number of calves ID’ed	–	11	3	4	5	10	5	8	7	15	9	6	12	11
Proportion of animals seen more than once in a season	0.43	0.60	0.72	0.93	0.85	0.95	0.67	0.72	0.71	0.75	0.77	0.46	0.73	0.94
Average number of days an animal was sighted in a season	1.57	2.00	2.61	4.28	4.22	6.64	3.10	3.43	4.10	2.84	2.58	1.69	4.98	7.72
Maximum number of days an animal was sighted in a season	4	5	13	11	13	16	10	10	15	9	7	5	25	42

**Fig. 3:**
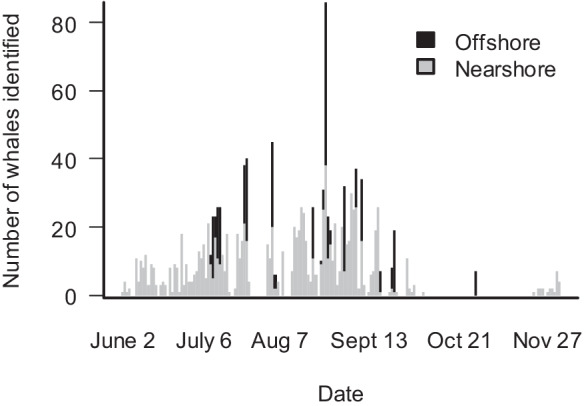
Number of whales identified for each day of the field season in 2015 by feeding area

The rate of identifying new individuals per sighting day was lower in 2015 compared to other years, but, unlike previous years, the number of identified whales may have reached an asymptote in 2015 (Fig. 2a). In addition, the time period during which data were collected in 2015 spanned the data collection periods of all other years (Fig. 2b). The daily count of identified animals in 2015 peaked on 25 August when 49% of all animals identified during the entire season were seen: 37 individuals in the nearshore area and 48 individuals in the offshore area (Fig. 3).

During 14 years of data collection, whales have been identified on a total of 112 offshore sighting days during which a total of 137 whales were identified. Five of those individuals (4%) have never been seen nearshore. All five individuals were seen in only one season, and two of them were seen on only one day. No demographic data were available for those individuals.

During 519 sighting days nearshore, a total of 252 individuals were identified, of which 120 (48%) were never seen offshore. Demographic data (sex, age, and reproductive status) were unknown for 23 identified nearshore-only animals, with the majority of them seen fewer than 3 years (*N* = 19). Calves that were not re-identified after their first foraging season (*N* = 51) represented 43% of the nearshore-only individuals. An additional 38 known-age, nearshore-only animals were identified past their first foraging season. Of those, sex has been determined for 18 of them (nine females and nine males), suggesting a 50:50 sex ratio at birth. Four nearshore-only animals were males of unknown age or reproductive status. The remaining four nearshore-only animals (3%) were reproductive females who exhibited high site fidelity and sighting probability, seen between 11 and 13 years of the 14-year study period. The remaining reproductive females in our dataset (*N* = 23) have been seen in both nearshore and offshore feeding areas. While not statistically significant, the four nearshore-only reproductive females had a slightly longer inter-calf interval (mean, 5.0; SD, 4.1 years) compared to females using the offshore area (mean, 3.9; SD, 1.5 years). An additional six reproductive females, as per the combined dataset used in Cooke et al. (2017), were not identified as such in our dataset, meaning we never had confirmed sightings of those individuals with a calf.

### Sighting location and age

Based on photo-ID data from 2002 to 2015, animals < 3.5 years old have never been sighted in the offshore feeding area (Online Resource 2). Of the 56 calves that were seen after their first foraging season, a total of 18 were eventually seen offshore. Of the 35 whales seen in their fourth feeding season (at roughly 3.5 years old), three were sighted offshore. The mean age of first detection offshore was 5.5 years (6th feeding season), with a maximum age of first offshore detection of 11.5 years (12th feeding season). One of seven individuals seen at the age of 12.5 years was never seen in the offshore area. That particular animal, a male, had been observed in the nearshore area every year except one, indicating a high degree of site fidelity to the nearshore foraging habitat.

**Fig. 4:**
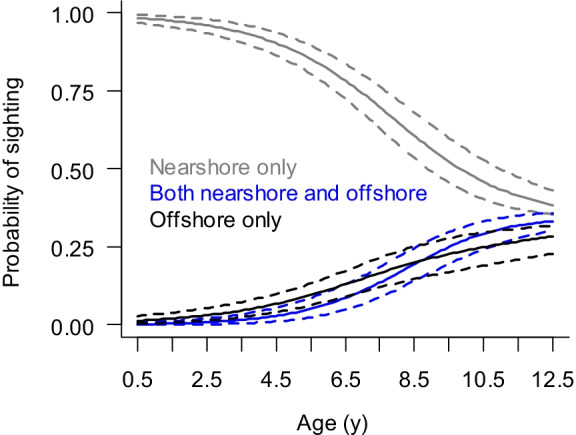
Posterior probability of sighting location as a function of age. Solid lines are mean estimates, and dashed lines are 95% posterior intervals

Overall, the probabilities of being identified in both areas or only in the offshore feeding area increased with age (Fig. 4). By the age of 12.5 years, animals had an almost equal probability of sighting only nearshore, both nearshore and offshore, or only offshore. While leave-one-out model weighting indicated models with nearshore prey energy and the proportion of nearshore energy from amphipods should be included in model averaging (Online Resource 3), overall parameter estimates for those variables became zero after averaging. In addition, inclusion of those prey variables did not change parameter estimates for the intercept or slope in the simplified model with age as the only predictor variable (Online Resource 4). Model weighting suggested effort should not be included in model averaging.

### Reproductive female sighting location and prey energy

**Fig. 5:**
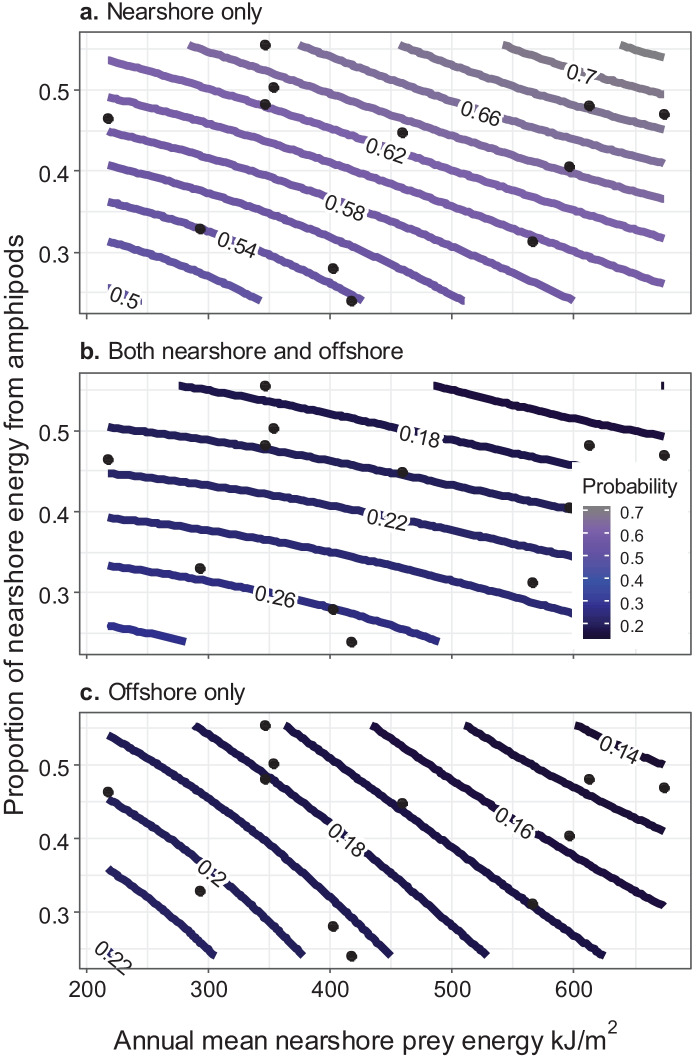
Probability of sighting pregnant females in the nearshore and/or offshore areas (see color spectrum and labels) as a function of annual mean nearshore prey energy and the proportion of nearshore energy from amphipods. Black points indicate annual values of prey metrics

**Fig. 6:**
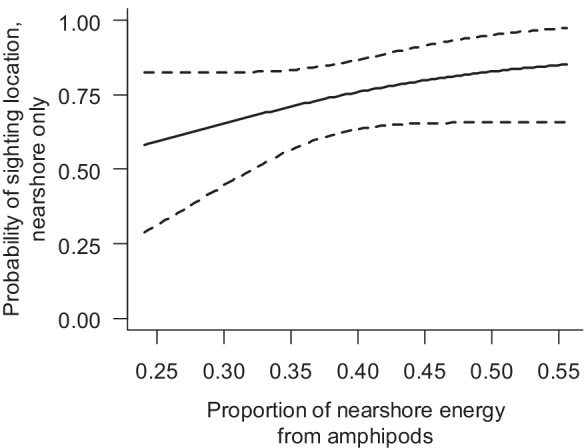
Probability a female that arrived with a calf is sighted only nearshore (compared to both nearshore and offshore) as a function of the proportion of nearshore energy from amphipods. All females arriving with calves are seen nearshore, while a subset of those animals is additionally seen in the offshore area. Solid line is the mean value with 95% posterior intervals as dashed lines

Mothers were identified with a total of 65 calves during the 14-year data collection period. Those females’ sighting locations during the previous summer represent utilization patterns during pregnancy. Based on photo-ID and whaling data, both eastern and western gray whales have a minimum inter-birth interval of 2 years (Rice & Wolman, 1971; Tyurneva et al., 2018). Weaning occurs during a calf’s first summer, so females are not simultaneously pregnant and nursing a calf. Pregnant females were seen more often nearshore with increased nearshore prey energy and an increase in the proportion of nearshore energy from amphipods (Fig. 5, Online Resources 3, 4, 5 and 6). During the following year when those females returned with their calf, the females were more likely to be sighted only in the nearshore area with increased proportion of nearshore energy from amphipods (Fig. 6, Online Resources 3, 4, 6, and 7). If a female that arrived with a calf was seen offshore, the calf was not with her, with the calf presumably still in the nearshore area. Overall, 23% of the identified females with a calf were seen both nearshore and offshore. The remaining 77% were sighted only nearshore. Model weighting suggests sighting location probabilities for successfully pregnant females and for females that arrived with a calf were not a function of effort. Nearshore and offshore prey energy were positively correlated with each other, as were nearshore and offshore proportions of prey energy from amphipods (Table 2, Online Resource 6).

**Table 2:** Correlations between year and prey metrics: nearshore and offshore prey energy (*E*_*n*_ and *E*_*o*_), proportion amphipod energy (*A*_*n*_ and *A*_*o*_), and prey biomass (*B*_*n*_ and *B*_*o*_)

	Year	*E*_n_	*E*_o_	*A*_n_	*A*_o_	*B*_n_	*B*_o_
Year	1.00	− 0.66	− 0.36	− 0.49	− 0.82	− 0.24	0.04
*E*_n_	− 0.66	1.00	0.71	− 0.04	0.32	0.85	0.54
*E*_o_	− 0.36	0.71	1.00	− 0.32	− 0.06	0.62	0.86
*A*_n_	− 0.49	− 0.04	− 0.32	1.00	0.70	− 0.42	− 0.60
*A*_o_	− 0.82	0.32	− 0.06	0.70	1.00	− 0.13	− 0.42
*B*_n_	− 0.24	0.85	0.62	− 0.42	− 0.13	1.00	0.66
*B*_o_	0.04	0.54	0.86	− 0.60	− 0.42	0.66	1.00

### Intra-seasonal sighting location and demographic group

Animals of known demographic group were sighted in the nearshore area on 106 days in 2015 and on 97 days for the combined 2002–2014 period (Online Resource 7). The number of sighting days for demographic groups was smaller for the offshore area (16 days in 2015 and 25 days combining 2002 through 2014 data) compared to nearshore. During any given day with whale sightings, average counts of identified whales of known demographic groups were higher nearshore (2015, 7.4 whales/day; 2002–2014, 13.4 whales/day) than offshore (2015, 4.5 whales/day; 2002–2014, 5.2 whales/day).

**Fig. 7:**
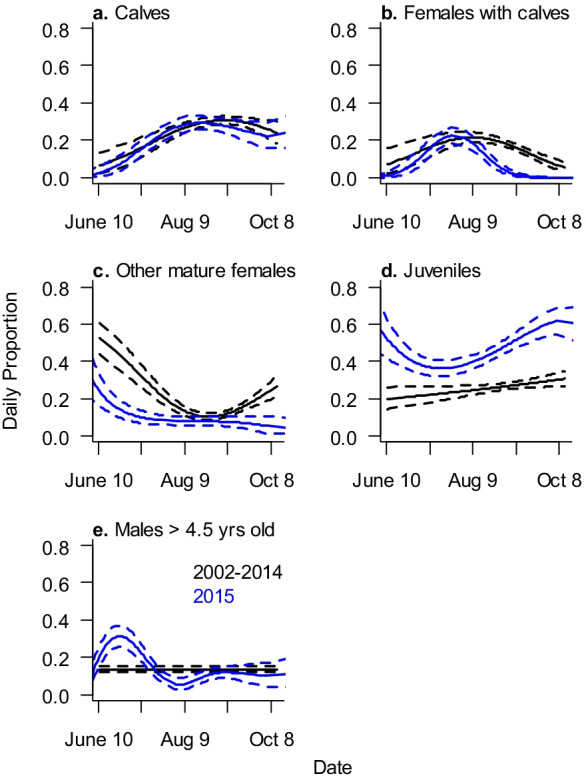
Daily proportion of different demographic groups in the nearshore area before 2015 (black) and during 2015 (blue). Solid lines are mean proportions, and dashed lines are 95% posterior intervals

The proportion of calves within the nearshore foraging group was roughly the same in 2015 and prior to 2015 (Fig. 7a). In 2015, females that arrived with calves moved out of the nearshore area earlier in the season compared to previous years (Fig. 7b, Online Resource 8). Reproductive females that were not seen with a calf constituted a higher proportion of the animals nearshore prior to 2015, while juveniles were a higher proportion during 2015 (Fig. 7c, d). While the proportion of males over 4.5 years of age in the nearshore feeding area fluctuated in 2015, the overall proportion of this demographic group was not different from previous years (Fig. 7e).

**Fig. 8:**
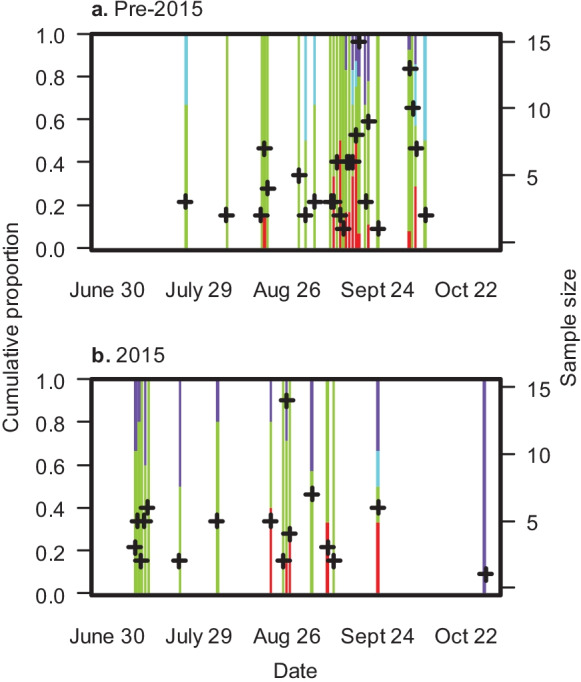
Cumulative daily proportion of different demographic groups in the offshore area **a** prior to 2015 and **b** during 2015. Colors indicate mothers separated from a calf (red), other reproductive females (green), juveniles (blue), and males > 4.5 years old (purple). “ + ” indicates sample size by day and is the observed number of animals within the given demographic categories

Sample sizes (number of sighting days) in the offshore area were too small to estimate changes in proportions of different demographic groups over the 2002–2014 and 2015 seasons. In general, reproductive females that were not seen with a calf in a given year made up the highest proportion of observed whales offshore both pre-2015 and during 2015 (pre-2015, 0.73; 2015, 0.64). Juveniles represented a smaller proportion of observed offshore whales (pre-2015, 0.06; 2015, 0.01). Males more than 4 years old made up a higher proportion of animals observed offshore in 2015 (0.24) compared to pre-2015 (0.09). The proportion of females that arrived with a calf but were eventually seen offshore was similar for both time periods (pre-2015, 0.12; 2015, 0.11). Overall, mothers who had recently weaned a calf constituted a higher proportion of the offshore whales as the season progressed (Fig. 8). The proportion of other reproductive females declined over the season, while males over 4.5 years old were constant (Fig. 8).

## Discussion

### Annual sighting summary

Prior to 2015, the number of identified animals did not asymptote with increased number of sighting days, indicating annual effort most likely did not capture all individuals in the area in a given year. However, an understanding of the observed asymptote in 2015 (when effort was increased) requires a quantified measure of effort for each day. For example, effort after October 1st (day 274) was reduced both spatially and temporally, meaning the likelihood of observing an unidentified whale was reduced. In addition, later in the season, whales might have been leaving the area before they were sighted. The earlier start date in 2015 most likely contributed to the lower daily discovery rate because animals were presumably still arriving. Future efforts to quantify the probability of missing a whale as well as arrival and departure times will require photo-ID effort to begin before whales arrive as well as spatial and temporal metrics of photo-ID effort for each day.

If the intent of photo-ID surveys focuses purely on identifying as many whales as possible, methodology could be simplified and targeted to a few specific days or locations during a season. For example, on 25 August, 2015, observers photographed 49% of the whales identified that season. The number of identified whales has also risen dramatically over the period of a few days in other years, so the ability to identify a large number of whales in a short period is not unique to 2015. Photographic effort targeting areas with the highest concentration of whales during the peak foraging season could continue to provide inter-annual data to calculate annual reproductive and survival rates. For example, an independent photo-ID study has focused effort at the mouth of Piltun Bay, and their annual capture rate (number of whales identified per days of effort) has been higher than the daily rates using the methodology in this study for all but 2 years since 2002 (Tyurneva et al., 2018). However, temporally or spatially limited methods will not provide the data needed to understand individual habitat use patterns between or within foraging seasons nor would such efforts provide as comprehensive data on individual reproduction or changes in body condition over a season. In essence, method simplification needs to be done with an understanding of the subsequent loss of information about physiology, movements, and reproduction.

### Sighting location and age

Gray whales appeared to increase utilization of the offshore feeding area as they aged, which is consistent with other studies (Bröker et al., 2020). After accounting for age, inter-annual differences in nearshore and offshore prey energy did not change the proportion of known-age animals seen in the nearshore area, the offshore area, or both. The reason for the shift offshore with age may be a combination of energetic needs, physiological foraging limitations, predator avoidance, and differences between nearshore and offshore prey energy. That combination may overshadow any relationship between inter-annual differences in prey energy and habitat use for younger animals.

Analysis of long-term photo-identification data of Sakhalin Island gray whales estimated age at sexual maturity at 9.0 years old (7.7–11.2 years old) (Bradford et al., 2010; Cooke, 2010), which is consistent with findings in eastern gray whales (Rice & Wolman, 1971). An additional female in our dataset successfully returned with a calf at the age of 10.5 years, making her the only successful reproductive female of known age in the dataset. Pregnant females in particular need to store extra energy to sustain themselves, complete the last months of pregnancy, and nurse a growing calf during their fasting period. Prey in the offshore feeding area provided a higher proportion of energy from amphipods and up to ten times higher energy content compared to prey composition in the nearshore feeding area (Blanchard et al., 2019; Maresh et al., 2022). Therefore, it seems reasonable to assume that prey energy would play a role in the shift in sightings to the higher prey energy offshore habitat as animals get larger with age and because reproductive females need to consume more prey energy for reproduction (Villegas-Amtmann et al., 2017).

The nearshore feeding area appears to be the preferred habitat for younger animals and mother–calf pairs. Given the high prey energy in the offshore area, why would some animals stay in the nearshore area? Killer whale predation pressure may account for the absence of younger animals and mother–calf pair sightings offshore (Bröker et al., 2020; Weller et al., 2018). Gray whales are observed to move closer to shore in the presence of killer whales, and young animals are particularly vulnerable to predation (Ford & Reeves, 2008). The long migration to shallow, coastal waters during the winter to give birth and breed may be a form of predator avoidance (Sumich, 2021). Smaller animals have lower energetic requirements, and nearshore prey energy may have been sufficient to meet their energetic needs. In addition, even with extremely high offshore prey energy, smaller animals may not be able to efficiently forage in the deeper offshore habitat, making the smaller animals’ net energy input lower when foraging in the offshore area compared to nearshore. Bioenergetics models suggest smaller animals do not benefit as much from the high offshore prey energy because they cannot physically process that amount of energy on a daily basis (McHuron et al., 2021). Therefore, the costs and risks of foraging offshore may have outweighed the benefits.

### Reproductive female sighting location and prey energy

Some sub-areas within the offshore area exhibit mean energy values ten times larger than what has been measured nearshore (Maresh et al., 2022). In fact, bioenergetics modeling results from McHuron et al. (2021) suggest pregnant females must utilize the offshore feeding area not only for successful reproduction but also for their own survival. However, one 12.5-year-old male and four reproductive females exhibited a high degree of site fidelity to the less energy-rich nearshore feeding area, as evidenced by repeated annual observations in that area coupled with no offshore sightings. So, the nearshore habitat must have contained enough nutrients to successfully support at least some larger whales. How do some reproductive whales successfully meet their survival and reproductive energetic needs when only foraging in the nearshore area? Nearshore foraging habitat might consist of ephemeral prey patches that are not effectively captured by annual benthic data collection methods coupled with simplified mean annual prey energy values. Furthermore, ocean conditions and safety concerns have limited sampling of nearshore benthic prey in waters of 7 m or less where gray whales have been regularly observed (Blanchard et al., 2019). Thus, some gaps in understanding total nearshore gray whale prey energy still remain.

Pregnant females were more likely to be seen only nearshore with increased nearshore prey energy, and both pregnant females and females with a calf had a higher sighting probability only nearshore with increased proportion nearshore energy from amphipods. Negative correlations with year indicated a decline in both prey energy and the proportion of energy from amphipods over time for both the nearshore and offshore areas. Results suggest females with high reproductive costs may still prefer the nearshore area, but declining nearshore energy may have increased their use of the offshore area. In fact, the majority of females who have been observed to successfully return with a calf used the offshore area both during the year of pregnancy and during the post-weaning period the following year. Some reproductive females exhibited high site fidelity to the nearshore feeding area, while a similar high inter-annual site fidelity to the offshore feeding area was not observed, suggesting some habitat preference to the nearshore area. However, it is possible that some reproductive females solely use the offshore area in both years and separate from their calves before or shortly after arriving at the foraging grounds, as some weaned calves were first identified in the nearshore area without their mothers. The majority of unaccompanied calves were identified later in the season, indicating these animals could have weaned before arriving later to the foraging grounds or before the mothers’ identification could be established. However, the mothers’ identities could have been missed in some years because of later survey start dates. Genetic testing could determine if such calves belong to as-yet-unidentified females that solely use the offshore area. Overall, our analysis showed that both the nearshore and offshore feeding areas are important to the population.

### Intra-seasonal sighting location and demographic group

An understanding of intra-seasonal habitat use patterns by different demographic groups can improve mitigation measures to reduce exposure to anthropogenic stressors, in particular for vulnerable demographic groups and groups that are more likely to affect population growth. In addition, habitat use patterns can be compared with bioenergetics models which suggest pregnant females are more likely to use the more energy-rich offshore area later in the season (McHuron et al., 2021). Sampling effort in 2015 allowed us to analyze intra-seasonal variability in proportions of some demographic groups during a year with high seismic activity and compare pre-2015 with 2015 results.

Changes in relative proportions of multiple groups are difficult to interpret since differences do not easily describe which groups are increasing or declining. Nevertheless, some conclusions can be drawn from comparing the proportions of different demographic groups utilizing the nearshore feeding area during 2015 compared to earlier years. Females that arrived with a calf were observed in the offshore area earlier in 2015, and none of them were seen nearshore after 26 August. None of the nearshore-only reproductive females was observed with a calf in 2015, which could explain the absence of females that arrived with calves remaining in the nearshore feeding area later in the 2015 season. Juveniles (1.5 to 4.5 years) represented a higher proportion of animals foraging nearshore in 2015 compared to previous years. The higher proportion of juveniles could be the result of high reproductive rates in the few years leading up to 2015, perhaps coupled with higher juvenile survival rates during that time. Alternatively, the lower proportion of reproductive females without a calf in the nearshore feeding area in 2015 could also result in the higher proportion of juveniles. In contrast to nearshore, reproductive females without a calf and males over 4.5 years generally represented a higher proportion of the offshore foraging group in 2015. Offshore prey biomass in 2015 was the highest recorded to date, but a comparatively low proportion of energy from amphipods resulted in 2015 offshore prey energy that was slightly above average across all years. Nearshore prey energy in 2015 was below the mean energy across all years. Therefore, changes in the demographic makeup of the animals observed in the nearshore and offshore feeding areas in 2015 could be an example of the older whales’ response to relative prey energy availability and/or acoustic disturbance that year that was not captured in the current analyses.

## Conclusions

This study aimed to address possible differences of western gray whale utilization of the nearshore and offshore feeding areas as a function of age, benthic prey energy, and demographic group. Our results indicated that younger whales remain nearshore, while the majority of older and reproductive animals utilize the nearshore and offshore areas with some flexibility based on prey energy. Intra-seasonal comparisons demonstrated that females arriving with their calves were no longer observed in the nearshore area earlier in 2015 compared to all previous years combined. Also, juveniles represented a higher proportion of whales seen in the nearshore area in 2015 compared to previous years.

This analysis aids in formulating additional analyses and determining future data collection needs to address several important population and ecosystem questions. For example, more information about the relationships between available prey energy in the nearshore and offshore areas and reproductive success can be gained through additional analyses of photo-ID data, including a quantification of the transfer functions between prey and body condition as well as body condition and reproduction (New et al., 2014). Also, photo-ID effort metrics, such as time searching for whales and environmental conditions while searching, could aid in movement probability models to determine the proportion of time each demographic group spends nearshore vs. offshore. Lastly, future analyses should endeavor to include anthropogenic acoustic metrics for both nearshore and offshore feeding areas. The results of this study provide the demographic and prey energy context for such studies.

## Supplementary Information

Below is the link to the electronic supplementary material.

Supplementary file1 (DOCX 42 KB)

Supplementary file2 (CSV 1 KB)

Supplementary file3 (DOCX 22 KB)

Supplementary file4 (CSV 0 KB)

Supplementary file5 (CSV 0 KB)

Supplementary file6 (CSV 0 KB)

Supplementary file7 (CSV 4 KB)

Supplementary file8 (DOCX 20 KB)
