# Supplementary material for: Western gray whales on their summer feeding ground off Sakhalin Island in 2015: who is foraging where?
Source: Environ Monit Assess. 2022 Oct 18;194(Suppl 1):738. doi: 10.1007/s10661-022-10022-x (PMC9579093; doi:10.1007/s10661-022-10022-x)
Supplement: Supplementary file 1 — Supplementary file1 (DOCX 42 KB) [file 10661_2022_10022_MOESM1_ESM.docx]

**Supplemental Material A. Sample Bayesian R code.**

**Data preparation**

To prepare data for analysis using ‘brms’ or ‘rjags,’ the “melt” function can be used on sighting data available in supplemental material to create one row per individual animal. Sighting data can be combined with explanatory variable data available in Table 1 and in supplementary material using the “merge” function.

**Library brms**

Seed values were changed for each model. All predictor variables were normalized. All brms simulations consisted of three independent chains with thinning of 30, a warmup of 30 samples, and a retained sample size of 5000 samples.

1. Estimating the proportion of known pregnant females sighted nearshore only, both nearshore and offshore, or offshore only (y) as a function of eight different variables (x): 0 (null), annual relative effort (number of nearshore days/total effort days, *D*), and nearshore and offshore prey biomass (*B_n_* and *B_o_*), prey energy (*E_n_* and *E_o_*), and proportion amphipod energy (*A_n_* and *A_o_*).

library(brms)

n.chains <- 3

n.thin <- 50

iterations <- 5000*n.thin

LP1 <- brm (y ~ x, data = Preg_Data,

family = categorical(link = "logit", refcat = NULL),

chains = n.chains,

iter = iterations,

warmup = n.thin,

thin = n.thin,

cores = n.chains,

seed = 3746)

LP1 <- add_criterion(LP1, c("loo", "waic"))

1. Estimating the proportion of females with a calf sighted nearshore only or both nearshore and offshore (y) as a function of eight different variables (x): null, *D*, *B_n_*, *B_o_*, *E_n_*, *E_o_*, *A_n_*, and *A_o_*.

library(brms)

n.chains <- 3

n.thin <- 50

iterations <- 5000*n.thin

LW1 <- brm (y ~ x, data = WithCalf_Data,

family = bernoulli(link = "logit"),

chains = n.chains,

iter = iterations,

warmup = n.thin,

thin = n.thin,

cores = n.chains,

seed = 374)

LW1 <- add_criterion(LW1, c("loo", "waic"))

1. Estimating the proportion of animals sighted nearshore only, both nearshore and offshore, or offshore only (y) as a function of nine different variables (x): null, *Age*, *D*, *B_n_*, *B_o_*, *E_n_*, *E_o_*, *A_n_*, and *A_o_*.

library(brms)

n.chains <- 3

n.thin <- 50

iterations <- 5000*n.thin

LA1 <- brm (y ~ x, data = Age_Data,

family = categorical(link = "logit", refcat = NULL),

chains = n.chains,

iter = iterations,

warmup = n.thin,

thin = n.thin,

cores = n.chains,

seed = 346)

LA1 <- add_criterion(LA1, c("loo", "waic"))

1. Model stacking

library(loo)

loo_model1 <- loo(model1)

loo_model2 <- loo(model2)

loo_model3 <- loo(model3)…

loo_model_weights(list(loo_model1, loo_model2, loo_model3,…))

**Library rjags**

In the case of polynomial variables of time, orthogonal transformation was used to reduce correlation between variables.

1. Estimating the proportion of five demographic groups (young of the year, females with young of the year, other reproductive females, juveniles, and males > 4 years old) sighted nearshore as a function of temporal variables: null and day of year (*t*, *t^2^*, *t^3^*). This example shows the final 2015 model.

library(rjags)

multinom2015.mod <-

"model{

# likelihood

for (i in 1:N){

NYoY[i] ~ dbin(p1[i], Ntot[i])

NMom[i] ~ dbin(p2[i], Ntot[i])

NReF[i] ~ dbin(p3[i], Ntot[i])

NJuv[i] ~ dbin(p4[i], Ntot[i])

NMal[i] ~ dbin(p5[i], Ntot[i])

# Linear predictors.

logit(p1[i]) <- alpha1 + (beta1a*DofY[i]) + (beta1b*DofY2[i]) + (beta1c*DofY3[i])

logit(p2[i]) <- alpha2 + (beta2a*DofY[i]) + (beta2b*DofY2[i])

logit(p3[i]) <- alpha3 + (beta3a*DofY[i]) + (beta3b*DofY2[i]) + (beta3c*DofY3[i])

logit(p4[i]) <- alpha4 + (beta4a*DofY[i]) + (beta4b*DofY2[i]) + (beta4c*DofY3[i])

p5[i] <- 1.0 - p1[i] - p2[i] - p3[i] - p4[i]

}

# priors

# YoY

alpha1 ~ dunif(-300,300)

beta1a ~ dunif(-300,300)

beta1b ~ dunif(-300,300)

beta1c ~ dunif(-300,300)

# Mom

alpha2 ~ dunif(-300,300)

beta2a ~ dunif(-300,300)

beta2b ~ dunif(-300,300)

# ReF

alpha3 ~ dunif(-300,300)

beta3a ~ dunif(-300,300)

beta3b ~ dunif(-300,300)

beta3c ~ dunif(-300,300)

# Juv

alpha4 ~ dunif(-300,300)

beta4a ~ dunif(-300,300)

beta4b ~ dunif(-300,300)

beta4c ~ dunif(-300,300)

}"

multinom2015.spec <- textConnection(multinom2015.mod)

# Orthogonal transformation of day of year, day of year^2, day of year^3

OrthDofY123 <- poly(t,3)

multinom2015.dataAll <- list( # 1st six vectors from data file.

NYoY = …,NMom = …,NReF = …,NJuv = …,NMal = …,Ntot = …,

DofY = OrthDofY123[,1],

DofY2 = OrthDofY123[,2],

DofY3 = OrthDofY123[,3],

N = …

)

# Initial values of unknown parameters.

multinom2015.inits <- list(

alpha1 = -10.0,beta1a = 0.0,beta1b = 0.0,beta1c = 0.0, # YoY

alpha2 = -10.0,beta2a = 0.0,beta2b = 0.0, # Mom

alpha3 = -10.0,beta3a = 0.0,beta3b = 0.0,beta3c = 0.0, # ReF

alpha4 = -10.0,beta4a = 0.0,beta4b = 0.0,beta4c = 0.0 # Juv

)

# Initialize the model.

chains <- 3

multinom2015.fit <- jags.model(

file = multinom2015.spec,

data = multinom2015.dataAll,

inits = multinom2015.inits,

n.chains = chains

)

# Let it run.

n.thin <- 30

iterations <- 5000*n.thin

burnin <- 100

multinom2015.samples <- coda.samples(

model = multinom2015.fit,

variable.names = c(

"alpha1","beta1a","beta1b","beta1c", # YoY

"alpha2","beta2a","beta2b", # Mom

"alpha3","beta3a","beta3b","beta3c", # ReF

"alpha4","beta4a","beta4b","beta4c"),# Juv

n.iter = iterations,

thin = n.thin)

1. Leave-one-out comparison from rjag posterior samples. First function calculates log posterior probability for each data point, specific to the best fitting 2015 model.

library(loo)

postsamp <- MCMCpstr(multinom2015.samples, type = 'chains')

Multinom2015_probout <- function(postsamp,DataFile,OrthDofY123){

# Posterior samples of parameters

# YoY

alpha1 <- postsamp[,1]; beta1a <- postsamp[,2]; beta1b <- postsamp[,3]; beta1c <- postsamp[,4]

# Mom

alpha2 <- postsamp[,5]; beta2a <- postsamp[,6]; beta2b <- postsamp[,7]

# ReF

alpha3 <- postsamp[,8]; beta3a <- postsamp[,9]; beta3b <- postsamp[,10]; beta3c <- postsamp[,11]

# Juv

alpha4 <- postsamp[,12]; beta4a <- postsamp[,13]; beta4b <- postsamp[,14]; beta4c <- postsamp[,15]

# Day of year (orthogonal transformation)

DofY = OrthDofY123[,1]; DofY2 = OrthDofY123[,2]; DofY3 = OrthDofY123[,3]

logprobout <- matrix(nrow = nrow(postsamp), ncol = nrow(DataFile))

for (I in 1:nrow(DataFile))){

NYoY <- DataFile[I,2]

NMom <- DataFile[I,3]

NReF <- DataFile[I,4]

NJuv <- DataFile[I,5]

NMal <- DataFile[I,6]

Ntot <- sum(DataFile[I,2:6])

logit_p1 <- alpha1 + (beta1a*DofY[I]) + (beta1b*DofY2[I]) + (beta1c*DofY3[I])

logit_p2 <- alpha2 + (beta2a*DofY[I]) + (beta2b*DofY2[I])

logit_p3 <- alpha3 + (beta3a*DofY[I]) + (beta3b*DofY2[I]) + (beta3c*DofY3[I])

logit_p4 <- alpha4 + (beta4a*DofY[I]) + (beta4b*DofY2[I]) + (beta4c*DofY3[I])

p1 <- exp(logit_p1)/(1.0+exp(logit_p1))

p2 <- exp(logit_p2)/(1.0+exp(logit_p2))

p3 <- exp(logit_p3)/(1.0+exp(logit_p3))

p4 <- exp(logit_p4)/(1.0+exp(logit_p4))

p5 <- 1.0 - p1 - p2 - p3 - p4

# Log likelihood.

PPPYoY <- (NYoY*p1) + ((Ntot - NYoY)*(1.0 - p1))

PPPMom <- (NMom*p2) + ((Ntot - NMom)*(1.0 - p2))

PPPReF <- (NReF*p3) + ((Ntot - NReF)*(1.0 - p3))

PPPJuv <- (NJuv*p4) + ((Ntot - NJuv)*(1.0 - p4))

PPPMal <- (NMal*p5) + ((Ntot - NMal)*(1.0 - p5))

PPP <- PPPYoY + PPPMom + PPPReF + PPPJuv + PPPMal

logprobout[,I] <- PPP

}

logprobout

}

# Relative effect size for each model.

rel_n_eff_1 <- relative_eff(exp(logpout_1), chain_id = rep(1,times=nrow(logpout _1)))

rel_n_eff_2 <- ...

# Leave-one-out calculations.

loo_model1 <- loo(logprobout_1, r_eff = rel_n_eff, is_method = "psis")

loo_model2 <- ...

# Comparing models.

loo_compare(x = list(loo_model1,loo_model2,...))

**Convergence and stationarity testing**

library(bayesplot)

library(MCMCvis)

library(coda)

# Lag-1 autocorrelation

mcmc_acf(brmsmodel, lags = 10)

# Visual inspection.

MCMCtrace(output.samples)

# Gelman and Rubin diagnostics

# Rhat < 1.05 for all parameters?

# n.eff > 10?

MCMCsummary(output.samples)

# Heidelberger and Welch diagnostics

# Stationarity

# Half width mean

heidel.diag(output.samples)
