# Supplementary material for: Western gray whales on their summer feeding ground off Sakhalin Island in 2015: who is foraging where?
Source: Environ Monit Assess. 2022 Oct 18;194(Suppl 1):738. doi: 10.1007/s10661-022-10022-x (PMC9579093; doi:10.1007/s10661-022-10022-x)
Supplement: Supplementary file 3 — Supplementary file3 (DOCX 22 KB) [file 10661_2022_10022_MOESM3_ESM.docx]

**Supplementary Table S1.** Model stacking weights for models estimating the probability of sighting location (*L_i_*, where *i* = nearshore only (*N*), both nearshore and offshore (*B*), or offshore only (*O*)) for different groups as a function of eight explanatory variables (*x*): nearshore and offshore prey energy (*E_n_* and *E_o_*), proportion amphipod energy (*A_n_* and *A_o_*), prey biomass (*B_n_* and *B_o_*), annual relative effort (number of nearshore days/total effort days, *D*), and age (*a*) from the equation $logit\left( L_{i\neq O} \right)=\beta_{0i}+\beta_{1\_i}x$ . Bold numbers indicate models that were included in final model averaging.

| *x* | Pregnant females | Females with calves | All known age |
| --- | --- | --- | --- |
| Null | 0 | **0.294** | **0.118** |
| *E_n_* | **0.405** | 0 | **0.196** |
| *E_o_* | 0 | 0 | **0.307** |
| *A_n_* | **0.595** | **0.704** | 0 |
| *A_o_* | 0 | 0.002 | 0 |
| *B_n_* | 0 | 0.001 | 0 |
| *B_o_* | 0 | 0 | 0 |
| *D* | 0 | 0 | 0 |
| *a* | NA | NA | **0.379** |

**Supplementary Table S2.** Posterior parameter distributions for models estimating the probability of sighting location (*L_i_*, where *i* = nearshore only (*N*), both nearshore and offshore (*B*), or offshore only (*O*)) for different groups as a function of eight explanatory variables (*x*): nearshore and offshore prey energy (*E_n_* and *E_o_*), proportion amphipod energy (*A_n_* and *A_o_*), prey biomass (*B_n_* and *B_o_*), annual relative effort (number of nearshore days/total effort days, *D*), and age (*a*) from the equation $logit\left( L_{i\neq O} \right)=\beta_{0i}+\beta_{1i}x$ . Explanatory variables were normalized before analysis.

| Group | Parameter | mean | se | CI_0.025_ | CI_0.975_ |
| --- | --- | --- | --- | --- | --- |
| Pregnant females | $\beta_{0B}$ | -0.18 | 0.65 | -1.49 | 1.07 |
|  | $\beta_{0O}$ | 0.38 | 0.60 | -0.79 | 1.58 |
|  | $\beta_{1E_{n}\_B}$ | -0.80 | 1.32 | -4.20 | 0.12 |
|  | $\beta_{1E_{n}\_O}$ | -0.64 | 1.10 | -3.54 | 0.29 |
|  | $\beta_{{1A}_{n}\_B}$ | -0.63 | 0.97 | -2.92 | 0.74 |
|  | $\beta_{1A_{n}\_O}$ | -1.25 | 1.28 | -3.83 | 0.00 |
| Females with calves | $\beta_{0B}$ | -0.36 | 0.67 | -1.54 | 0.91 |
|  | $\beta_{1A_{n}\_B}$ | -1.62 | 1.36 | -4.26 | 0.00 |
| All known ages | $\beta_{0B}$ | -4.16 | 0.48 | -5.17 | -3.28 |
|  | $\beta_{0O}$ | -6.06 | 0.79 | -7.77 | -4.65 |
|  | $\beta_{1a\_B}$ | 5.66 | 0.92 | 3.94 | 7.52 |
|  | $\beta_{1a\_O}$ | 8.32 | 1.22 | 6.11 | 10.89 |
|  | $\beta_{1E_{n}\_B}$ | 0.00 | 0.00 | 0.00 | 0.00 |
|  | $\beta_{1E_{n}\_O}$ | 0.00 | 0.00 | 0.00 | 0.00 |
|  | $\beta_{1E_{o}\_B}$ | 0.00 | 0.00 | 0.00 | 0.00 |
|  | $\beta_{1E_{o}\_O}$ | 0.00 | 0.00 | 0.00 | 0.00 |
